# Genetic diversity of drug-resistant *Mycobacterium tuberculosis* clinical isolates from Khuzestan province, Iran

**DOI:** 10.1186/s13568-022-01425-7

**Published:** 2022-07-04

**Authors:** Pejman Bakhtiyariniya, Azar Dokht Khosravi, Mohammad Hashemzadeh, Mohammad Savari

**Affiliations:** 1 Infectious and Tropical Diseases Research Center, Health Research Institute, Ahvaz Jundishapur University of Medical Sciences, Ahvaz, Iran; 2 Department of Microbiology, Faculty of Medicine, Ahvaz Jundishapur University of Medical Sciences, Ahvaz, Iran; 3 Iranian Study Group on Microbial Drug Resistance, Tehran, Iran

**Keywords:** Tuberculosis, *Mycobacteriumtuberculosis*, Drug resistance, MIRU-VNTR typing, Genetic diversity

## Abstract

The emergence of drug-resistant strains of the *Mycobacterium tuberculosis* (MTB) has challenged tuberculosis control programs. So far, few studies using the 24-locus mycobacterial interspersed repetitive unit variable number tandem repeats (MIRU-VNTR) have investigated the genetic diversity of MTB in Iran. This study aimed to determine the genetic diversity of MTB isolates resistant to first-line anti-tuberculosis drugs using 24-locus MIRU-VNTR in southwestern Iran. Out of 6620 MTB clinical isolates, 29 resistant isolates to one or more isoniazid, rifampin, and ethambutol were detected using drug susceptibility testing by the proportional method. The manual 24-locus MIRU-VNTR was used to determine the MTB resistant isolates’ phylogenetic relationship. MIRU-VNTR*plus* web application tools were applied to analyze the associated data. Using 24-locus MIRU-VNTR, 13.8% of isolates (n = 4) were distributed in two clusters, and the remaining 86.2% (n = 25) showed a unique pattern. Four clonal complexes were observed in the minimum spanning tree based on the double-locus variant. Most isolates belonged to Delhi/CAS (34.5%, 10/29) and NEW-1 (24.1%, 7/29) sub-lineages, followed by EAI and LAM with a frequency of 6.9% (2/29) and 3.5% (1/29), respectively. Eight isolates (27.6%) did not match any genotype in the database. The 24-locus MIRU-VNTR showed a high discriminatory power; however, the 15-locus and 12-locus set analyses were more discriminative. Our study revealed a high degree of genetic diversity among drug-resistant MTB isolates, which could be interpreted as the low rate of person-to-person transmission in this region. The 15-locus MIRU-VNTR would be recommended for preliminary genotyping of drug-resistant MTB.

## Introduction

Despite the availability of effective drugs and comprehensive global action under the guidance of the World Health Organization (WHO) in recent decades, tuberculosis (TB) caused by the *Mycobacterium tuberculosis* (MTB) is still considered a public health problem, and it is at the top causes of human death (MacNeil et al. 2020). The latest WHO report (2021) estimated that about two billion people are infected with MTB, and 10 million new TB cases globally occur each year. These facts demonstrate a large reservoir for TB and express the importance of worldwide disease control. The emergence of drug-resistant forms of TB, including mono-resistant (i.e., resistant to any of the isoniazid, rifampin, ethambutol, or pyrazinamide drugs—known as first-line drugs) and multidrug-resistant (MDR; i.e., simultaneously resistant to both isoniazid and rifampin) has made disease control more complicated and more necessary (Chakaya et al. 2021).

According to the Iranian Center for Tuberculosis and Leprosy Control (2021), Iran has significantly controlled TB in the last three decades. In terms of total incidence, MDR incidence, and mortality, Iran has far lower rates than the global and regional averages (WHO 2019). Due to the tremendous cultural similarities between the border residents of Iran and neighboring countries, there are significant border crossings, which have increased people’s exposure and may jeopardize the national achievements of TB control (Tavakoli 2017). This situation requires the best tools of epidemiological studies to follow the chain of disease transmission.

Molecular epidemiology is the study of disease distribution in a human population utilizing molecular typing methods based on repetitive DNA elements on the genome as markers to identify the strain (Shi et al. 2018; Joseph et al. 2013). Epidemiologists have used molecular tools to assess risk factors related to recent transmission, track the dynamics of infection transmission, and distinguish endogenous reactivation from exogenous reinfection. So, these tools can be of great help in researching and controlling tuberculosis (Shi et al. 2018). Restriction fragment length polymorphism (RFLP) based on IS*6110* (van Embden et al. 1993) was the first method used for molecular typing of MTB and was considered the gold standard for many years. However, due to its limitations and disadvantages, including labor-intensiveness, difficult interpretation of results, and insufficient discriminatory power for isolates with low IS*6110* copy numbers, it was gradually replaced by polymerase chain reaction (PCR)-based methods (Supply et al. 2006).

Analysis of variable numbers of tandem repeats (VNTRs) of mycobacterial interspersed repetitive units (MIRUs) (Supply et al. 2001) is now commonly used for genotyping strains of MTC. MIRUs are short genetic elements (50–100 bp) that are often found as tandemly repeated sequences, and the loci containing them are scattered throughout the intergenic regions of the MTB genome. Isolates of MTB are different in the number of repeats at various MIRU loci (Ramazanzadeh and McNerney 2007). The standardized MIRU-VNTR genotyping system, consisting of 15- and 24-locus sets, has been proposed for epidemiological and phylogenetical studies, respectively (Gauthier et al. 2015). The advantages of this method compared to IS*6110*-based RFLP analysis are faster performance, applicability to non-purified DNA extracts (Supply et al. 2006), lower economic costs (Shi et al. 2018), more discriminatory power for isolates with low IS*6110*-copy numbers (Joseph et al. 2013), more straightforward implementation technically, higher reproducibility at intra- and interlaboratory levels (Gauthier et al. 2015), and portability of results due to the generation of digital profiles in the form of numerical codes (Ramazanzadeh and McNerney 2007).

Khuzestan, located in southwestern Iran, ranks fifth among the provinces regarding TB incidence based on the Iranian Center for Tuberculosis and Leprosy Control report (2021). Due to huge industries, large commercial ports, and active border terminals, a considerable population travels in and out of the province at the national and international levels annually. Therefore, the control of communicable infectious diseases, especially TB, is at the top of the attention of policymakers and executives of the health sector in the province. Given the importance of investigating the molecular epidemiology of resistant strains, this study was conducted with two objectives. The primary purpose was to determine the genetic diversity of drug-resistant MTB clinical isolates from Khuzestan province by the 24-locus MIRU-VNTR method. In addition, the discriminatory power of the 24-locus set was compared with variants of 15- and 12-locus sets of the mentioned method.

## Materials and methods

### Bacterial isolates

A total of 29 drug-resistant MTB isolates obtained from patients with clinical symptoms of pulmonary TB were included in this study. The isolates were collected from 6620 sputum samples sent to the Regional Tuberculosis Reference Laboratory of Khuzestan province, Iran, from April 2014 to March 2021. The isolates were phenotypically identified as MTB using the following procedures: microscopy after acid-fast staining, growth on Löwenstein–Jensen (LJ) culture medium, and biochemical tests such as nitrate reductase, niacin accumulation, and heat-stable catalase (Kent and Kubica 1985). The *M. tuberculosis* H37Rv (ATCC^®^ 27294) was used as the reference strain.

### Drug susceptibility testing

According to the WHO instructions (2018), the susceptibility of isolates to first-line anti-TB drugs was tested using the proportion method on the LJ medium. In brief, critical concentrations of 0.2, 40, and 2 µg/ml were prepared for isoniazid, rifampin, and ethambutol (Sigma-Aldrich, Germany), respectively. Twenty-nine cultures that showed 1% or more growth on a drug-containing medium compared with the growth on a control without the same drug were considered resistant. The *M. tuberculosis* H37Rv was used for quality control.

### DNA extraction and molecular identification

A simple boiling procedure was used to extract the genomic DNA from resistant isolates and the reference strain with few modifications to the previously described method (Blackwood et al. 2000). Briefly, a few fresh colonies of each isolate was harvested from the LJ medium and dissolved in 500 µl TE (Tris-EDTA) buffer (pH 8.0). Bacterial suspensions were boiled twice for 15 min at 100 °C with a 5-min cooling interval at − 21 °C and subsequently centrifuged at 13,000 rpm for 5 min. The supernatants were transferred to new microtubes and stored at − 21 °C until used as templates in PCR amplification.

To definitively identify the isolates, a 130 bp fragment of the IS*6110* element was amplified by specific forward (5′-CTCGTCCAGCGCCGCTTCGG-3′) and reverse (5′-CCTGCGAGCGTAGGCGTCGG-3′) primers (Khosravi et al. 2012). The reaction mixtures were prepared in a final volume of 25 µl, containing 12.5 µl of Taq DNA polymerase 2× Master Mix RED (AMPLIQON, Denmark), 10 pmol of each primer, and 5 µl of the genomic DNA (65 ng/µl). PCR amplification was carried out in a thermal cycler (Eppendorf 6333, Hamburg, Germany) under the following condition: initial denaturation at 95 °C for 5 min, followed by 35 cycles of 95 °C for 30 s, 63 °C for 30 s, 72 °C for 30 s, and a final extension at 72 °C for 5 min.

The PCR products were separated by electrophoresis on a 1.2% agarose gel (EMD Millipore, Billerica, MA, USA) stained with the SYBR^®^ Safe DNA Gel Stain (Thermo Fisher Scientific), and the DNA bands were visualized using a gel documentation system (Uvidoc HD6, UVITEC, Cambridge, UK). The isolates with a band pattern similar to the reference strain were confirmed as MTB.

### MIRU-VNTR genotyping

Manual 24-locus MIRU-VNTR typing was performed for drug-resistant MTB isolates as described by Supply (2005). Aliases assigned to loci are as follows in order of their conventional numbers: MIRU 02, Mtub04, ETRC, MIRU 04, MIRU 40, MIRU 10, MIRU 16, Mtub21, MIRU 20, QUB-11b, ETRA, Mtub29, Mtub30, ETRB, MIRU 23, MIRU 24, MIRU 26, MIRU 27, Mtub34, MIRU 31, Mtub39, QUB-26, QUB-4156, MIRU 39.

Each locus was amplified separately using specific unlabeled previously designed primers (Supply et al. 2006). In brief, 2 µl of template DNA (65 ng/µl) was added to the simplex PCR mixture consisting of 10 µl of Taq DNA polymerase 2× Master Mix RED (AMPLIQON, Denmark), 8 pmol of each primer, 0.5–1.5 mM additional MgCl_2_ for some loci, and distilled water up to a final volume of 20 µl. Negative controls contained all reaction components except DNA. The reactions were performed in a Bio-Rad (USA) thermal cycler with the following program: 15 min initial denaturation at 95 °C followed by 40 cycles of 94 °C for 1 min, annealing (depending on MIRU loci) for 1 min, 72 °C for 1.5 min, and terminated by a final extension at 72 °C for 10 min. The most appropriate annealing temperatures obtained in this study for MIRU loci were: 61, 59, 59, 62.5, 61, 63, 65.5, 56.5, 51, 59, 59, 55, 63, 59, 63.5, 60, 61, 63, 63.5, 58, 59, 59, 41, and 64 °C, respectively.

The PCR products of each locus were separated by electrophoresis on a 2% agarose gel (EMD Millipore, Billerica, MA, USA) stained with the SYBR^®^ Safe DNA Gel Stain (Thermo Fisher Scientific) and by using a gel documentation system (Uvidoc HD6, UVITEC, Cambridge, UK). The sizes of the amplicons were determined by the software PyElph 1.3 in comparison with the control strain (H37Rv) and standard 100- and 50-bp DNA size markers (Pavel and Vasile 2012). Next, by referring to the allele-calling table available at http://www.miru-vntrplus.org, the sizes of amplicons were converted to MIRU-VNTR alleles representing the number of tandem repeats of the loci for each isolate.

### Data analysis

Numerical data resulting from genotyping were uploaded to the MIRU-VNTR*plus* web application (https://www.miru-vntrplus.org/MIRU/index.faces) in three formats compatible with 12-, 15-, and 24-locus MIRU-VNTR sets. After selecting MIRU-VNTR as the genotyping method and categorical as the distance measure in the tool “Identification by similarity search,“ the lineages of isolates were predicted by assigning the closest and best matching strains among the complete reference database. A dendrogram based on the UPGMA (Un-weighted Pair Group Method with Arithmetic Mean) algorithm and a minimum spanning tree (MST) were created using the same application (Allix-Béguec et al. 2008). Isolates with identical genotypes were grouped in a cluster.

The discriminatory powers of the MIRU-VNTR method and the allelic diversity of each MIRU locus were assessed using the Hunter and Gaston Discriminatory Index (HGDI). The formula for calculating the index is as follows:

$$\text{D}=1-\frac{1}{N\left(N-1\right)}{\sum }_{j=1}^{S}{n}_{j}({n}_{j}-1)$$

Explicitly, *N* indicates the total number of strains in the sample, *S* represents the total number of types, and *n*_*j*_ is the number of strains belonging to the *j*th type (Hunter and Gaston 1988). Accordingly, the discriminatory power of loci was considered low (HGDI ˂ 0.3), moderate (0.3 ≤ HGDI ≤ 0.6), or high (HGDI ˃ 0.6) (Sola et al. 2003).

The significance of the association between the two classified data was examined by Chi-squared or Fisher’s exact tests. For this purpose, a *p*-value < 0.05 was statistically significant.

## Results

This study included 29 MTB isolates resistant to one or more first-line anti-TB drugs identified and collected from 11 counties of Khuzestan province, Iran, since 2014. The isolates belonged to 28 Iranian patients (2 isolates belonged to one patient obtained in different years) with pulmonary TB and were isolated from sputum samples. The demographic information was available only for 22 patients. Patients ranged in age from 31 to 70 years (median 36 and mean 39 years). Males accounted for 90.9% of cases with a male to female ratio of 10 (20/2). One of the patients had a history of imprisonment.

All isolates were confirmed to belong to the MTB by amplifying the IS*6110* gene fragment. Drug susceptibility test revealed that 28% (n = 8), 14% (n = 4), and 10% (n = 3) of isolates were mono-resistant to rifampin, ethambutol, and isoniazid, respectively. There were 10 MDR isolates (34% of the total), and 3 were co-resistant to rifampin and ethambutol. Only one isolate showed triple resistance to rifampin, ethambutol, and isoniazid. Altogether, resistance to rifampin, isoniazid, and ethambutol alone or in combination was found in 76% (n = 22), 48% (n = 14), and 28% (n = 8) of isolates, respectively.

**Fig. 1:**
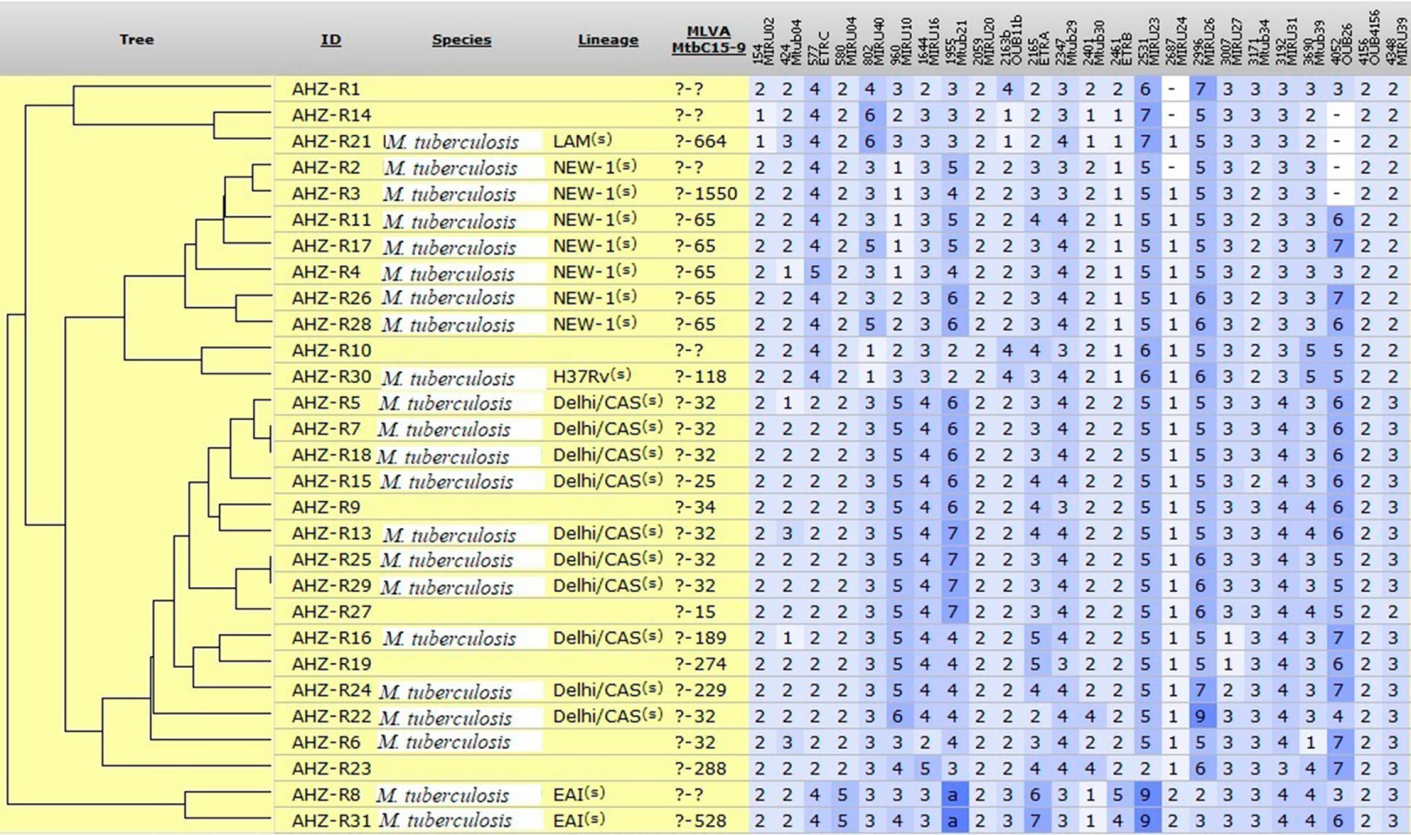
Phylogenetic tree of 29 MTB resistant isolates in Khuzestan province, Iran, based on 24-locus MIRU-VNTR data. Using the UPGMA algorithm, the dendrogram was generated by the features available in the MIRU-VNTR *plus* web application

Analysis based on 24-locus MIRU-VNTR identified 27 distinct genotypes, including 2 clusters (each consisting of 2 isolates, 13.8% of the total) and 25 unique patterns (86.2%) among 29 drug-resistant isolates (Fig. 1). Of these unique genotypes, 8 (27.6% of the total) were not assignable to the sub-lineages in the MIRU-VNTR *plus* database and were therefore termed “Unknown.“ Two isolates belonging to one patient (AHZ-R16 and AHZ-R19) obtained in two different years were not classified in a cluster and showed two unique patterns.

The majority of the isolates (34.5%, n = 10) belonged to the East-African-Indian (L3) (EAI) lineage, followed by the Euro-American (L4) (EA) and Indo-Oceanic (L2) (IO) lineages with 31.0% (n = 9) and 6.9%, (n = 2) respectively. Among the isolates of EA lineage, the NEW-1 sub-lineage was more common (24.1%, n = 7), and in terms of prevalence, it was ranked second after Delhi/CAS sub-lineage from EAI lineage (34.5%, n = 10). Table 1 compares the frequency of each lineage between MDR and non-MDR isolates. The most common sub-lineage among MDR isolates was Delhi/CAS, while in non-MDR isolates, Delhi/CAS and NEW-1 sub-lineages with equal prevalence account for more than 60% of cases. However, this difference was not statistically significant. Isolates AHZ-R7, AHZ-R18, AHZ-R25, and AHZ-R29, grouped in two clusters, belonged to patients from four different counties, although the first three isolates were MDR.

**Table 1:** Distribution of lineages among MDR and non-MDR MTB isolates (n = 29)

Lineages	Sub-lineages	No. (%) of MDR isolates (n = 10)	No. (%) of non-MDR isolates (n = 19)	*p*-value
East-African-Indian (EAI)	Delhi/Central Asian (Delhi/CAS)	4 (40.0%)	6 (31.6%)	0.650198
Euro-American (EA)	NEW-1 Latin American-Mediterranian (LAM) H37Rv-like	1 (10.0%) 1 (10.0%) 0 (0.0%)	6 (31.6%) 0 (0%) 1 (5.3%)	0.544809 0.557072 0.317311
Indo-Oceanic (IO)	East-African-Indian (EAI)	1 (10.0%)	1 (5.3%)	0.317311
Unknown		3 (30.0%)	5 (26.2%)	0.317311
Total		10 (100.0%)	19 (100%)	

Calculation of the Hunter and Gaston index showed that the discriminatory power of 24-locus MIRU-VNTR was high (HGDI = 0.835). The allelic polymorphism at different loci was relatively varied, ranging from 7 (locus Mtub21) to only 1 allele (loci MIRU 20 and QUB-4156). Table 2 presents the discriminatory power calculated for each locus using HGDI. Loci Mtub21, QUB-26, MIRU 10, ETRA, MIRU 16, and MIRU 26 had the highest (HGDI > 0.6), loci MIRU 27, MIRU 04, and MIRU 02 had low (HGDI < 0.3), and loci MIRU 20 and QUB-4156 had no discriminatory power. The rest of the loci were moderately discriminative.

**Table 2:** Allelic diversity and discriminatory power of different MIRU-VNTR loci among 29 MTB isolates

Locus	Allelic polymorphism	Allelic diversity (HGDI)	Discriminatory power	24-locus VNTR	15-locus VNTR	12-locus VNTR	19-locus VNTR^a^	Modified 15-locusVNTR^a^
MIRU 02	2	0.067	Low	*		*		
Mtub04	3	0.369	Moderate	*	*		*	*
ETRC	3	0.549	Moderate	*	*		*	*
MIRU 04	2	0.067	Low	*	*	*		
MIRU 40	5	0.424	Moderate	*	*	*	*	*
MIRU 10	6	0.771	High	*	*	*	*	*
MIRU 16	4	0.613	High	*	*	*	*	*
Mtub21	7	0.855	High	*	*		*	*
MIRU 20	1	0.0	Low	*		*		
QUB-11b	4	0.419	Moderate	*	*		*	*
ETRA	6	0.707	High	*	*		*	*
Mtub29	2	0.443	Moderate	*			*	
Mtub30	3	0.360	Moderate	*	*		*	*
ETRB	4	0.569	Moderate	*			*	*
MIRU 23	5	0.470	Moderate	*		*	*	
MIRU 24	2	0.318	Moderate	*		*	*	
MIRU 26	6	0.611	High	*	*	*	*	*
MIRU 27	3	0.197	Low	*		*		
Mtub34	2	0.468	Moderate	*			*	
MIRU 31	2	0.512	Moderate	*	*	*	*	*
Mtub39	5	0.581	Moderate	*	*		*	*
QUB-26	5	0.820	High	*	*		*	*
QUB-4156	1	0.0	Low	*	*			
MIRU 39	2	0.512	Moderate	*		*	*	*

^a^Analysis based on 19-locus and modified 15-locus MIRU-VNTR sets were performed explicitly in this study

**Fig. 2:**
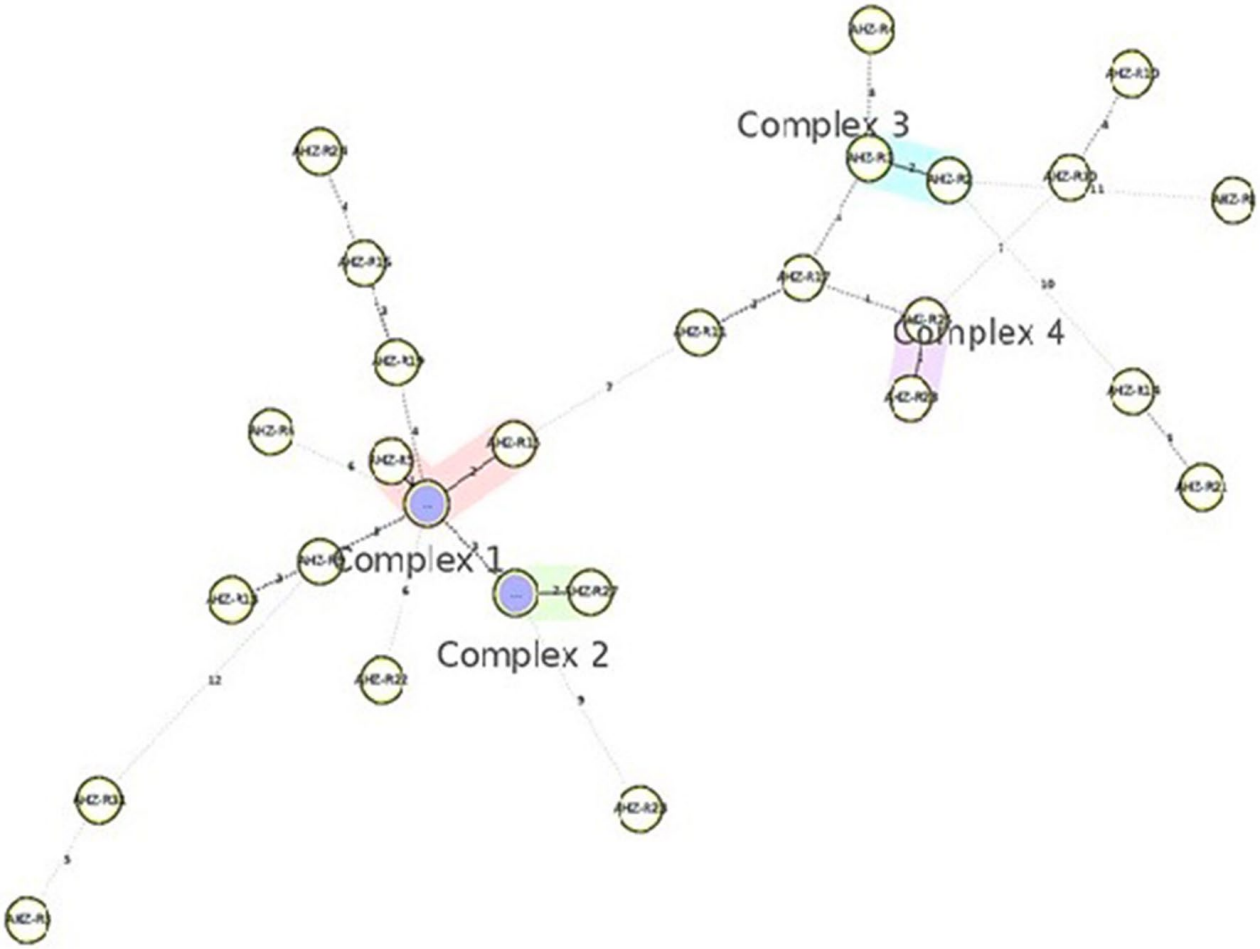
Minimum spanning tree (MST) of 27 genotypes of MTB calculated based on MIRU-VNTR data for 29 isolates from Khuzestan province, Iran, created by the features available in the MIRU-VNTR *plus* web application

Utilizing MIRU-VNTR data, an MST was constructed in which genotypes of isolates were linked based on double-locus variants (Fig. 2). Nine genotypes (corresponding to 11 isolates) were grouped into 4 clonal complexes, leaving 18 singleton patterns. Clonal complexes 1 and 2 contained three and two genotypes of the Delhi/CAS sub-lineage, respectively, and each of the clonal complexes 3 and 4 encompassed two genotypes of the NEW-1 sub-lineage.

The analysis of the 24-locus set was compared with 12-, 15-, 19-, and modified 15-locus sets to investigate the discriminatory power of various locus sets in the MIRU-VNTR method (Table 3). The composition of each locus set is given in Table 2. The composition of the 19-locus set was obtained by removing low-discriminative loci from the 24-locus set. The modified 15-locus set was created by replacing the two high-power (ETRB and MIRU 39) instead of the two low-power (MIRU 04 and QUB-4156) loci in the conventional 15-locus set. For the isolates tested, the analysis of the conventional 15-locus set had the highest discriminatory power, although the highest number of isolates without the assigned genotype (n = 11; 37.9%) also occurred with this set. The following ranks of discriminatory power belonged to 12-locus and 24-locus sets with a slight difference. Even though the 19-locus set had the least discriminatory power, it generated the highest genotypic differentiation (23/29, 79.3%).

**Table 3:** Comparison of genotype production ability and discriminatory power of different locus sets of MIRU-VNTR method for MTB isolates (n = 29)

Sub-lineages	24-locus	15-locus	12-locus	19-locus	Modified 15-locus
Delhi/CAS	10	5	10	12	12
NEW-1	7	5	2	6	5
EAI	2	2	2	2	2
LAM	1	2	2	1	1
H37Rv-like	1	1	–	1	1
Cameroon	–	2		–	1
Uganda I	–	1	1	–	–
Haarlem	–	–	4	1	–
X	–	–	1	–	–
Unknown	8	11	7	6	7
HGDI	0.835	0.943	0.867	0.798	0.810

## Discussion

The epidemiological significance of drug-resistant TB cases and their substantial treatment costs to the health sector led this study to focus on MTB isolates resistant to first-line anti-TB drugs. Although 34% of resistant isolates under study were MDR, a recent systematic review showed that the prevalence of MDR isolates in Khuzestan province (6.1%) was lower than the national average (6.3%) from 2013 to 2020 (Khademi and Sahebkar 2021). The rate of rifampin resistance was higher than isoniazid and ethambutol in our clinical isolates. This finding agrees with the results of studies in some other regions of Iran (southern, western, central, and southeastern provinces) (Pourakbari et al. 2016) and the previous report from Khuzestan province (Sirous et al. 2018). Rifampin resistance develops rapidly, mainly as monotherapy, and according to WHO guidelines (2010), it should always be prescribed in combination with other anti-TB drugs. The TB treatment success rate in Khuzestan province has been reported at 94.2% (Alavi et al. 2012), higher than in some other provinces (Ayatollahi et al. 2014; Khazaei et al. 2016). On the other hand, the TB treatment failure rate in Khuzestan (3.1%) was in the range of the mentioned reports. Nevertheless, the higher resistance of the isolates to rifampin may be due to any impairment in the administration, usage, or monitoring of the patient’s treatment regimen.

Different studies have demonstrated the significant resolution power of the 24-locus MIRU-VNTR typing system to genetically differentiate MTB isolates for investigating TB transmission and the clonal expansion and diversity of particular strains or lineages (Bouklata et al. 2015). Previously, studies were performed to determine the genetic diversity of MTB isolates from Khuzestan. These studies, which were sometimes multicentric, either used the 12-loci MIRU-VNTR method, did not focus specifically on resistant isolates, or determined the genotypes of isolates by other techniques. Therefore for the first time in this province, the present study used the 24-locus MIRU-VNTR method to investigate resistant MTB isolates’ genetic diversity and genotypic differentiation.

The results showed that 29 isolates were genotypically divided into two binary clusters and 25 unique patterns. The multiplicity of unique genotypes was interpreted as relatively high genetic diversity. Genetic diversity, which reflected genomic differences between isolates, indicated relative phylogenetic distances within and between lineages. Numerous studies have provided evidence showing the impact of genetic diversity on various aspects of virulence, including transmission (Coscolla and Gagneux 2014). Rates of MTB transmission are inferred by comparing genotypic clustering between isolates from a given epidemiological setting (Spuy et al.  2003). Thus, the epidemiological inference from the low number of clusters was that the current transmission rate has been negligible. Consequently, the person-to-person transmission may not have played a significant role in spreading drug-resistant MTB isolates in the province. A study aimed at genotyping MDR isolates collected from seven provinces of Iran (including Khuzestan) by the 12-locus MIRU-VNTR obtained similar results (Khosravi et al. 2017). The development of clinical drug resistance in TB is classified as acquired (= secondary) resistance when drug-resistant mutants are selected following ineffective treatment or primary resistance when a patient is infected with a resistant strain (Johnson et al. 2006). So, patients carrying isolates under study had secondary drug resistance rather than primary type. “Unknown” isolates (27.6%) did not match the MIRU-NVTR*plus* database’s genotypes, suggesting that new types may be developing in the province and country. Using methods with higher resolution, such as whole-genome sequencing, or at least the combination of MIRU-VNTR and spoligotyping in future studies, will help differentiate and classify the unknown isolates.

The most common sub-lineage identified in the present study was Delhi/CAS, to which 34.5% of the isolates belonged. The CAS sub-lineage of MTB from the L3 lineage dominates the Eastern Hemisphere, but its highest prevalence is reported in South and West Asia and some East African countries. This distribution view probably stems from the long history of trade relations and migration within and between these regions (Couvin et al. 2019). For example, of the total isolates surveyed recently, 59.1% in northern India (Sharma et al. 2017), 53.4% in Pakistan (Bakuła et al. 2019), and 41.8% in Iraq (Ahmed et al. 2014) belonged to the Delhi/CAS sub-lineage. In reviewing the articles, we did not find an independent study from Afghanistan, but Delhi/CAS was the most prevalent (36.4%) sub-lineage among Afghan refugee patients living in Iran (Riyahi Zaniani et al. 2017). The Delhi/CAS prevalence varies in different parts of Iran, although it seems to be more common in the central provinces. A systematic review study considered this sub-lineage the second most prevalent (19.2%) circulating genotype in Iran (Hadifar et al. 2021). As Khuzestan province is adjacent to Iraq, the study conducted by Ahmed et al. (2014) can reasonably compare our findings. In addition to religious commonalities, part of the population of Khuzestan province has linguistic and ethnic similarities with the Iraqi people. Strong social, economic, and religious ties that lead to frequent cross-border travel may justify the superiority of the Delhi/CAS sub-lineage over the MTB population in Iraq and Khuzestan. In addition, 70% of the Iraqi isolates that belonged to the Delhi/CAS sub-lineage were MDR, and although it was much higher than ours, it showed the high tendency of Delhi/CAS isolates to drug resistance.

Isolates belonging to the NEW-1 sub-lineage came in second with 24.1% in this study. It was rare to find a report of this subset of the EA (L4) lineage in Iranian studies until about a decade ago. Since then, researchers from various regions, especially in the north and the capital, have repeatedly reported it (Kochkaksaraei et al. 2019; Azimi et al. 2018). The NEW-1 sub-lineage was identified as the dominant (21.9%) circulating genotype in Iran by a systematic review of studies of the last decade (Hadifar et al. 2021). In a multicenter study covering nine Iranian provinces, the NEW-1 sub-lineage made up the most (41.2%) resistant isolates (Mousavi et al. 2020). Recently, a hypothesis has been put forward about the origin and dispersal of this sub-lineage: its ancestor originated in China centuries ago, entered Iran through the invasion of the Mongol Empire about 800 years ago, and gradually evolved into NEW-1. It remained endemic in Iran until the flux of migrants, especially Afghan refugees, has led to a marked increase in the prevalence of this sub-lineage and its significant association with drug resistance in Iran, Afghanistan, and Pakistan over the past 20 years (Mokrousov et al. 2017). In our study, 58.6% of the isolates belonged to Delhi/CAS and NEW-1, so the overall superiority of the two sub-lineages was consistent with previous findings. The reversal of their position compared to other regions of Iran may be due to the smaller number of isolates in the present study and the exclusive focus on resistant isolates.

In addition to the 24-locus analysis, we also analyzed 12-locus, conventional 15-locus, 19- locus, and modified 15-locus sets, as mentioned earlier. The last two sets were explicitly designed for this study. Unexpectedly, 15-locus and even 12-locus analyzes were more discriminative than 24-locus analysis for our isolates (HGDI = 0.943 and 0.867 compared to 0.835, respectively). Numerous studies in Iran have reported the discriminatory power of 15-locus MIRU-VNTR analysis to be very high (HGDI ≥ 0.98) and desirable for the genotypic differentiation of MTB (Kochkaksaraei et al. 2019; Azimi et al. 2018; Mousavi et al. 2020). Nevertheless, a study in China calculated the HGDI of 12-, 15-, and 24-locus analysis for 668 isolates at 0.925, 0.996, and 0.998, respectively (Shi et al. 2018), which is inconsistent with the present study for the 24-locus analysis. Analysis of the 19-locus set led to the highest lineage assignation (23/29; 79.3%) but had the weakest discriminatory power (HGDI = 0.798). The latter finding may seem confusing and deceptive at first glance, but a closer look at the HGDI formula components will solve the problem. According to this formula, the smaller the *n*_*j*_s (i.e., the number of strains assigned to each sub-lineage), the higher the index values (i.e., the discriminatory power).

Allelic diversity expresses the power of loci in discriminating against different genotypes. We compared the results of the present study on allelic diversity with an Iranian study from Isfahan province (Zaniani et al. 2018) and two foreign studies from China and Morocco (Shi et al. 2018; Bouklata et al. 2015) that performed a 24-locus MIRU-VNTR for genotyping of MTB isolates. QUB-26, MIRU 26, and Mtub21 had high discriminatory power, exactly like the three mentioned studies. The high discriminatory power of MIRU 10 in this study was consistent with studies in Isfahan and Morocco (HGDI = 0.7 and 0.61, respectively). MIRU 16 and ETRA showed high discriminatory power only in this study and Isfahan (HGDI = 0.61 and 0.67), while the former was considered weak locus in China (HGDI = 0.172) and Morocco (HGDI = 0.23). On the other hand, loci with low discriminatory power are probably conserved and do not significantly affect the differentiation of MTB isolates. MIRU 04 revealed low discriminatory power in all four studies. In our study, the low discriminatory power of MIRU 20 and MIRU 27 was similar to Isfahan (HGDI = 0.3 and 0) and China (HGDI = 0.030 and 0.175). In the present study and China, MIRU 02 was low-discriminative (HGDI = 0.067 and 0.015, respectively). QUB-4156 showed low power in this study, whereas this locus was moderately powerful in the other three studies.

In the current study, we had some limitations. There was a small sample size, although we included all the resistant MTB isolates identified in the last seven years in the region. Second, it was not possible to access the demographic information of some patients from whom the isolates were obtained. This limitation prevented us from accurately and thoroughly examining the relationship between genotyping results and the location of all patients.

In conclusion, our findings revealed that the MIRU-VNTR method in various settings could be an effective tool for molecular epidemiology studies. Although 24-locus MIRU-VNTR showed good discriminatory power, the 15-locus analysis had the highest HGDI and appeared applicable at least to the preliminary genotyping in this region. The predominant genotype among the resistant MTB isolates of Khuzestan province belonged to Delhi/CAS and New-1 sub-lineages. This finding is similar to circulating sub-lineages in other parts of Iran and neighboring countries (including Iraq, adjacent to the province). The high frequency of unique patterns, small clusters, and low population in each clonal complex indicated a high genetic diversity of drug-resistant MTB isolates in Khuzestan province. The interpretation of current results was that drug resistance mainly occurred following miss-drug used rather than person-to-person transmission. Thus, to promote anti-TB programs in the province, it seems necessary to intensify accurate and continuous monitoring of the prescription and consumption of first-line anti-TB drugs.

## Data Availability

All data generated or analyzed during this study are included in the present published article and are available from the corresponding author upon reasonable request.

## Abbreviations

MTB: *Mycobacterium* *tuberculosis*
TB: Tuberculosis
MDR: Multidrug resistant
MIRU-VNTR: Mycobacterial interspersed repetitive unit variable number tandem repeats
WHO: World Health Organization
EAI: East-African-Indian
EA: Euro-American
IO: Indo-Oceanic
PCR: Polymerase chain reaction
RFLP: Restriction fragment length polymorphism
LJ: Löwenstein–Jensen
MST: Minimum spanning tree
UPGMA: Un-weighted pair group method with arithmetic mean
HGDI: Hunter and Gaston Discriminatory Index
